# Disruption of mitochondria-associated ER membranes impairs insulin sensitivity and thermogenic function of adipocytes

**DOI:** 10.3389/fcell.2022.965523

**Published:** 2022-09-09

**Authors:** Chih-Hao Wang, Chen-Hung Wang, Pen-Jung Hung, Yau-Huei Wei

**Affiliations:** ^1^ Graduate Institute of Biomedical Sciences, China Medical University, Taichung, Taiwan; ^2^ Center for Mitochondrial Medicine and Free Radical Research, Changhua Christian Hospital, Changhua City, Taiwan; ^3^ Institute of Clinical Medicine, National Yang Ming Chiao Tung University, Taipei, Taiwan

**Keywords:** mitochondria-associated ER membranes, insulin resistance, type 2 diabetes, reactive oxygen species, white adipocytes, brown adipocytes, thermogenesis

## Abstract

The prevalence and healthcare burden of obesity and its related metabolic disorders such as type 2 diabetes (T2D) are increasing rapidly. A better understanding of the pathogenesis of these diseases helps to find the therapeutic strategies. Mitochondria and endoplasmic reticulum (ER) are two important organelles involved in the maintenance of intracellular Ca^2+^ and ROS homeostasis. Their functional defects are thought to participate in the pathogenesis of insulin resistance or T2D. The proper structure and function of the mitochondria-associated ER membranes (MAMs) is required for efficient communication between the ER and mitochondria and defects in MAMs have been shown to play a role in metabolic syndrome and other diseases. However, the detailed mechanism to link MAMs dysfunction and pathogenesis of insulin resistance or T2D remains unclear. In the present study, we demonstrated that the proteins involved in .MAMs structure are upregulated and the formation of MAMs is increased during adipogenic differentiation of 3T3-L1 preadipocytes. Disruption of MAMs by knocking down GRP75, which is responsible for connecting ER and mitochondria, led to the impairment of differentiation and ROS accumulation in 3T3-L1 preadipocytes. Most importantly, the differentiated 3T3-L1 adipocytes with GRP75 knockdown displayed inactivation of insulin signaling pathway upon insulin stimulation. Moreover, GRP75 knockdown impaired thermogenesis and glucose utilization in brown adipocytes, the adipocytes with abundant mitochondria that regulate whole-body energy homeostasis. Taken together, our findings suggest that MAMs formation is essential for promoting mitochondrial function and maintaining a proper redox status to enable the differentiation of preadipocytes and normal functioning such as insulin signaling and thermogenesis in mature adipocytes.

## Introduction

Increasing evidence has supported a causative relationship between mitochondrial dysfunction and the pathogenesis of type 2 diabetes (T2D) and insulin insensitivity (Petersen et al., 2004; Mogensen et al., 2007; Wang and Wei, 2020). It has been reported that the tissues of mice and human subjects with insulin insensitivity or T2D display lower expression levels of respiratory enzyme complexes or proteins essential for mitochondrial biogenesis, impaired respiratory function or defects in the β oxidation of fatty acids. Decline in mitochondrial bioenergetic function is commonly observed in insulin-responsive tissues (muscle and adipose tissues) of diabetic mice or T2D patients. Moreover, more profound decline in mitochondrial function would lead to more severe hyperglycemia and insulin insensitivity of diabetic patients and mouse models (Lim et al., 2006; Park and Lee, 2007; Pravenec et al., 2007; Wang et al., 2013). These observations strongly support the concept that mitochondrial dysfunction is one of the major etiological factors for T2D and insulin resistance.

In addition to mitochondrial defects, abnormality of endoplasmic reticulum (ER) is also associated with T2D. Chronic ER stress and dysregulation of unfolded protein response (UPR) are involved in the mechanisms underlying obesity-induced insulin insensitivity. However, alleviation of ER stress by treatment of diabetic mice with 4-phenyl butyric acid (4-PBA) and taurine-conjugated ursodeoxycholic acid (TUDCA) was found to improve the metabolic abnormalities in these mice (Ozcan et al., 2006). Mitochondria and ER are two intracellular organelles that play a key role in the maintenance of cellular homeostasis. Mitochondria and ER are physically and functionally interconnected, sharing some important cellular functions such as Ca^2+^ homeostasis. Alterations in the crosstalk between mitochondria and ER could result in the development of T2D.

Mitochondria-associated ER membranes (MAMs) are the contact sites between the mitochondrial outer membrane and ER (Patergnani et al., 2011). The MAMs refer to a bridge region of two membranes where the distance between the two organelles is less than 25 nm (Csordas et al., 2006). The physical interactions between both organelles depend on complementary membrane proteins, which tether the two organelles together at specific sites. For example, the voltage-dependent anion channel (VDAC) of the outer mitochondrial membrane interacts with the inositol 1,4,5-triphosphate receptor (IP3R) on the ER through the molecular chaperone glucose-regulated protein 75 (GRP75), allowing Ca^2+^ transport from the ER to mitochondria. Recently, mitofusin 2 (Mfn2) was discovered as a direct ER-mitochondria tether, which also regulates the interaction and Ca^2+^ transport between the two organelles (Yang et al., 2020). It was further demonstrated that the MAMs architecture involves a large number of proteins with various functions (Yang et al., 2020).

In the present study, we found that the formation of MAMs is increased during adipogenic differentiation of 3T3-L1 cells. The disruption of MAMs by knock-down of GRP75, a protein involved in MAMs formation, led to the impairment of differentiation and mitochondrial dysfunction although there was a compensatory upregulation of mitochondrial biogenesis. Ultimately, increase of the ROS level due to imbalance of the antioxidant system impaired the insulin sensitivity and thermogenic function in mature white and brown adipocytes with GRP75 deficiency, respectively.

## Materials and methods

### Culture of preadipocytes

3T3-L1 and WT-1 mouse brown preadipocytes, which were obtained from the laboratory of Dr. Yu-Hua Tseng at Joslin Diabetes Center, Harvard Medical School were grown in Dulbecco’s Modified Eagle’s Medium (DMEM) (Gibco, Invitrogen, Grand Island, NY) supplemented with 10% fetal bovine serum (FBS), 100 units/ml penicillin, 100 μg/ml streptomycin sulfate and 0.25 mg/ml amphotericin B (Biological Industries, Kibbutz Beit Haemek, Israel) at 37°C containing 5% CO_2_. The cells were sub-cultured every 3 days when reaching 60%–70% of confluence. In all experiments, 3T3-L1 and brown preadipocytes were used for differentiation to mature adipocytes within 5 passages.

### Adipogenic differentiation of preadipocytes

For white adipogenic differentiation of 3T3-L1, 2 days after confluence (day 0), 3T3-L1 cells were grown at 37°C in the differentiation medium composed of 10% DMEM, 0.25 μM dexamethasone, 0.5 mM 3-isobutyl-1-methylxanthine, and 10 μg/ml insulin containing 10% CO_2_ for 3 days. After induction of differentiation, the adipocytes were cultured in 10% DMEM containing 1 μg/ml insulin for the first 2 days and then in the fresh 10% DMEM without insulin in the following 2 days. All the experiments were conducted by using the adipocytes 7 days after differentiation.

For brown adipocyte differentiation, 2 days after reaching confluence (day 0), WT-1 mouse brown preadipocytes were grown at 37°C for 3 days in the differentiation medium composed of 10% DMEM, 5 μM dexamethasone, 0.5 mM 3-isobutyl-1-methylxanthine, 125 μM indomethacin, 1 nM T_3_ and 0.5 μg/ml insulin containing 10% CO_2_. After induction of differentiation, the adipocytes were cultured in 10% DMEM containing 1 nM T_3_ and 0.5 μg/ml insulin every 2 days. All the experiments were conducted by using the brown adipocytes 7 days after differentiation.

### GRP75 knockdown in 3T3-L1 and brown preadipocytes

The lentiviruses containing two different small hairpin RNA (shRNA) constructs of the GRP5 gene (GRP75 KD #1 and #2) and random sequences (CTL KD) were obtained from the RNAi Core Facility at Academia Sinica, Taipei, Taiwan. To achieve the maximum efficiency of knockdown of GRP5, the 3T3-L1 and brown preadipocytes infected with lentivirus for 24 h were selected by addition of 1 μg/ml puromycin to the culture medium for another 6 days. The targeting sequences of CTL KD and GRP75 KD in the pLKO.1 plasmid are listed in Table 1.

**TABLE 1:** The oligonucleotide sequences of shRNA used in this study

	Sequence (5’→3′)
CTL KD	CAAATCACAGAATC GTCGTAT
GRP75 KD #1	GCT​GGA​GAC​AAC​AAA​CTT​CTA
GRP75 KD #2	GCT​GTT​ATG​GAG​GGC​AAA​CAA

### Immunoprecipitation assay for the assess*ment of* mitochondria-associated endoplasmic reticulum membranes *formation*

IP3R1 and GRP75 were precipitated from the total lysate of 3T3-L1 cells before (day 0) and 7 days after adipogenic differentiation using a Mag Sepharose Xtra kit (GE Healthcare Life Sciences, Buckinghamshire, United Kingdom) according to the manufacturer’s instructions. Briefly, 1 mg total protein lysate of 3T3-L1 cells was incubated with specific primary antibodies (α-IP3R1 or α-GRP75) at 4°C overnight, which was followed by incubating the mixture with 20 μl of immunocapture beads at 4°C for 4 h. After washing with the TBST buffer (50 mM Tris-HCl, 150 mM NaCl, and 0.1% Tween-20, pH 7.4), the target protein and its interacting proteins were eluted by 2% SDS and then analyzed by Western blotting.

### RNA extraction and gene expression analysis

Total cellular RNA was extracted with chloroform after addition of the TRIzol Reagent (Invitrogen), precipitated with isopropanol and then dissolved in DEPC-H_2_O. An aliquot of 5 µg RNA was reverse-transcribed into cDNA using a Ready-to-Go RT-PCR kit (GE Healthcare Life Sciences) at 42°C for at least 16 h. Q-PCR analysis was performed using the SYBR master kit according to the manufacturer’s instructions. The mRNA expression levels of the target genes were normalized against the attachment region binding protein (ARBP). The sequences of the primer pairs are listed in Table 2.

**TABLE 2:** The oligonucleotide sequences of primers used in this study

Gene	Sequence (5’→3′)
ARBP_F	TTT​GGG​CAT​CAC​CAC​GAA​AA
ARBP_R	GGA​CAC​CCT​CCA​GAA​AGC​GA
PPARγ2_F	TCA​GCT​CTG​TGG​ACC​TCT​CC
PPARγ2_R	ACC​CTT​GCA​TCC​TTC​ACA​AG
AP2_F	AAG​GTG​AAG​AGC​ATC​ATA​ACC​CT
AP2_R	TCA​CGC​CTT​TCA​TAA​CAC​ATT​CC
Adiponectin_F	GGA​GAG​AAA​GGA​GAT​GCA​GGT
Adiponectin_R	CTTTCCTGCCAGGGGTTC
PGC1α_F	CCC​TGC​CAT​TGT​TAA​GAC​C
PGC1α_R	TGC​TGC​TGT​TCC​TGT​TTT​C
AKT2_F	CCC​TCA​AGT​ATG​CCT​TCC​AG
AKT2_R	ACC​ACA​TCT​CTC​GAG​TGC​AA
IRS1_F	GTA​GAG​AGC​CAC​CAG​GTG​CTT​GT
IRS1_R	CTG​GAG​TAT​TAT​GAG​TCG​AGA​AGA​AG
NRF1_F	CAA​CAG​GGA​AGA​AAC​GGA​AA
NRF1_R	GCA​CCA​CAT​TCT​CCA​AAG​GT
NRF2_F	AGG​TTG​CCC​ACA​TTC​CCA​AAC​AAG
NRF2_R	TTG​CTC​CAT​GTC​CTG​CTC​TAT​GCT
TFAM_F	GTC​CAT​AGG​CAC​CGT​ATT​GC
TFAM_R	CCC​ATG​CTG​GAA​AAA​CAC​TT
MnSOD_F	GAC​CCA​TTG​CAA​GGA​ACA​A
MnSOD_R	GTA​GTA​AGC​GTG​CTC​CCA​CAC
Catalase_F	CCT​TCA​AGT​TGG​TTA​ATG​CAG​A
Catalase_R	CAA​GTT​TTT​GAT​GCC​CTG​GT
IP3R1_F	CGT​TTT​GAG​TTT​GAA​GGC​GTT​T
IP3R1_R	CAT​CTT​GCG​CCA​ATT​CCC​G
GRP75_F	ATG​GCT​GGA​ATG​GCC​TTA​GC
GRP75_R	GCA​CCC​TTG​ATT​GCT​TCT​GAT​G
VDAC1_F	CCC​ACA​TAC​GCC​GAT​CTT​GG
VDAC1_R	GCT​GCC​GTT​CAC​TTT​GGT​G
MCU_F	GAG​CCG​CAT​ATT​GCA​GTA​CG
MCU_R	CGA​GAG​GGT​AGC​CTC​ACA​GAT
MCUR1_F	CTC​AGC​CTG​TCT​GCT​AAG​TGC
MCUR1_R	GAG​AGC​GAT​TTC​CTG​CTG​C
MICU1_F	CTT​AAC​ACC​CTT​TCT​GCG​TTG​G
MICU1_R	AGC​ATC​AAT​CTT​CGT​TTG​GTC​T
UCP1_F	CTG​CCA​GGA​CAG​TAC​CCA​AG
UCP1_R	TCA​GCT​GTT​CAA​AGC​ACA​CA
ADRB3_F	TCT​CTG​GCT​TTG​TGG​TCG​GA
ADRB3_R	GTT​GGT​TAT​GGT​CTG​TAG​TCT​CG
C/EBPβ_F	CAA​CCT​GGA​GAC​GCA​GCA​CAA​G
C/EBPβ_R	GCT​TGA​ACA​AGT​TCC​GCA​GGG​T
PRDM16_F	CAG​CAC​GGT​GAA​GCC​ATT​C
PRDM16_R	GCGTGCATCCGCTTGTG
FAS_F	GGA​GGT​GGT​GAT​AGC​CGG​TAT
FAS_R	TGG​GTA​ATC​CAT​AGA​GCC​CAG
GLUT1_F	CAG​TTC​GGC​TAT​AAC​ACT​GGT​G
GLUT1_R	GCC​CCC​GAC​AGA​GAA​GAT​G

### Protein extraction and western blot analysis

Cells were incubated with the lysis buffer containing 50 mM HEPES (pH 7.4), 4 mM EDTA, 2 mM EGTA, 1 mM Na_2_VO_3_, 1 mM NaF, 1% Triton X-100, and protease inhibitors (Roche) at 4°C for 20 min and the lysate was centrifuged at 10,000 g for 30 min at 4°C. An aliquot of 50 μg proteins was subjected to electrophoresis on a 12% SDS-PAGE gel and then transferred onto a piece of the PVDF membrane (Pall Corporation, Port Washington, NY). After blocking, the membrane was hybridized with the indicated primary antibodies and the corresponding HRP-conjugated secondary antibody. Finally, the protein bands were visualized by an ECL chemiluminescence reagent (Perkin-Elmer Life Sciences) and the band intensities were determined using a Luminescence Imaging System Model LAS4000 (GE Healthcare Life Sciences, Chicago, IL). All the data were normalized against an internal control, actin or β tubulin.

The primary antibodies against IP3R1, p-AKT (ser473), total AKT, MCU and β tubulin were obtained from Cell Signaling Technology (Danvers, MA), the antibody against GRP75 was purchased from Proteintech (Rosemont, IL), antibodies against VDAC1 and actin were obtained from Merck Millipore (Billerica, MA).

### Measurement of insulin response

After adipogenic differentiation, the mature 3T3L1 adipocytes were incubated with a serum-free medium for 4 h and then treated with (insulin-stimulated group) or without (basal group) 100 nM insulin for 30 min to activate insulin signaling pathway. For the measurement of insulin response of cells, the ratio of p-AKT/Akt was determined using Western blot analysis.

### Measurement of ROS production

After adipogenic differentiation, the levels of mitochondrial superoxide anion and intracellular hydrogen peroxide in adipocytes were measured by the fluorescent dyes MitoSOX Red and 2′,7′-dichlorodihydrofluorescein diacetate (DCFH_2_-DA), respectively (Wang et al., 2013; Wu et al., 2018). The adipocytes were incubated with 5 μM MitoSOX Red or 80 μM DCFH_2_-DA in the medium at 37°C in the dark for 30 min. After washing with PBS, the relative fluorescence intensity of MitoSOX or DCF in 10,000 cells per sample was determined on a flow cytometer (Model EPICS XL-MCL, Beckman-Coulter, Miami, FL). The excitation and emission wavelengths used are 510/580 nm and 488/535 nm for mitoSOX and DCF, respectively.

### Measurement of cytosolic Ca^2+^

The cytosolic Ca^2+^ level was detected by a cell-permeant calcium dye, Fluo-4 AM. Mature adipocytes were incubated with 1 μM Fluo-4 AM, at 37°C in the dark for 30 min. After washing with PBS, the levels of calcium-bound Fluo-4 in cytosol of adipocytes were determined by a flow cytometer using the excitation wavelength at 494 nm and emission wavelength at 506 nm.

### Detection of glucose uptake

Glucose uptake was measured by using 2-[N-(7-nitrobenz-2-oxa-1,3-diazol-4-yl)amino]-2-deoxy-d-glucose (2-NBDG), a fluorescent glucose analog, according to the previous protocol (Yamada et al., 2007). Adipocytes were grown in a serum-free medium for 4 h and the medium was then replaced by a KRH buffer (KRP buffer containing 20 mM HEPES, pH 7.2). The adipocytes were stimulated with (insulin-stimulated group) or without (basal group) addition of 100 nM insulin. After incubation for 30 min, a suitable volume of 50 μM 2-NBDG in KRH buffer was directly added to allow the cells to uptake 2-NBDG for 20 min. Insulin was placed totally for 50 min. After washing with PBS, the 2-NBDG uptake was determined on a flow cytometer using the excitation wavelength at 465 nm and emission wavelength at 540 nm.

### Statistical analysis

Statistical analyses were performed using the Microsoft Excel 365 statistical package and the data are presented as means ± SEM of the results obtained from 3 or more independent experiments. The significance level of the difference between control and experimental groups was determined by Student’s *t* test. A difference is considered significant when *p* value < 0.05 (*), <0.01 (**) or <0.005 (***).

## Results

### Increase of mitochondria-associated endoplasmic reticulum membrane formation during adipogenic differentiation

**FIGURE 1:**
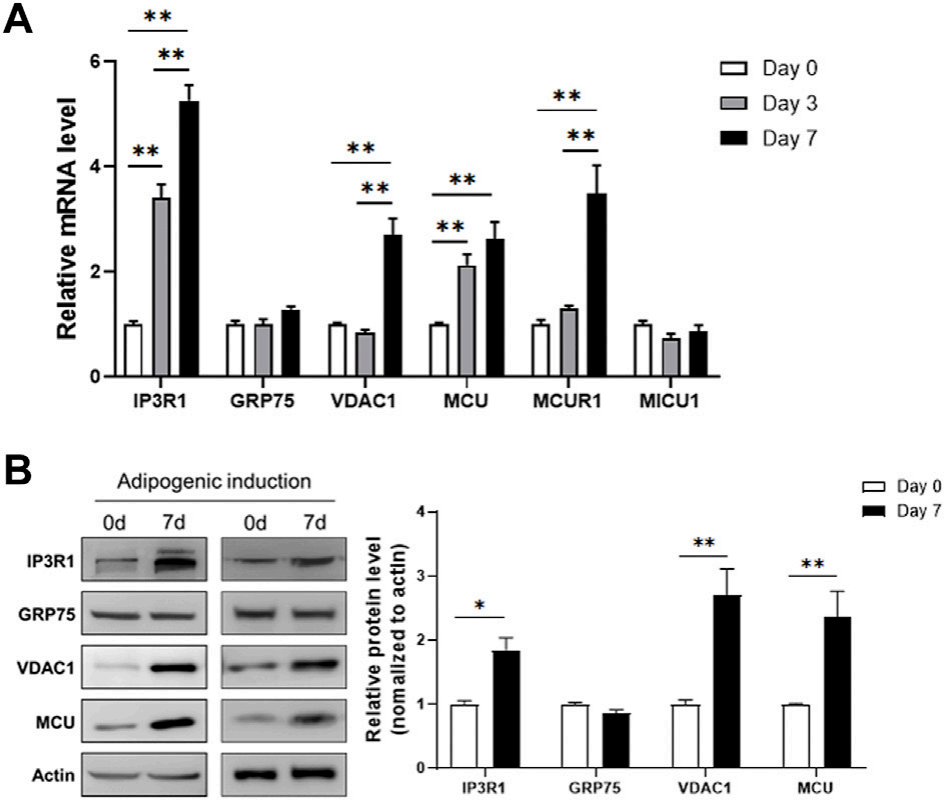
Increased expression levels of MAMs-related genes after adipogenic differentiation. **(A)** The mRNA levels of genes involved in the formation of MAMs structure on day 0, 3, 7 after adipogenic differentiation of 3T3-L1 preadipocytes. N = 3 in each group. **(B)** The protein levels of genes involved in the formation of MAMs structure before (Day 0) and 7 days after adipogenic differentiation of 3T3-L1 preadipocytes. N = 3 in each group. Data are presented as means ± SEM.; *p < 0.05; **p < 0.01.

To examine the change of MAM formation during differentiation of 3T3-L1, we collected the RNA samples at different time points (day 0, 3, 7) after adipogenic induction and measured the expression levels of genes involved in the MAMs structure. The results showed that the channel protein IP3R1 in the ER, and the channel proteins in mitochondrial outer and inner membranes, VDAC1 and MCU, respectively, were all upregulated during adipogenic differentiation (Figure 1A). However, GRP75, the linker between IP3R1 and VDAC1 to connect ER and mitochondria, was not changed during differentiation (Figure 1A). In addition, we extracted total cellular proteins before and after adipogenic differentiation of 3T3-L1 preadipocytes. Similar to the results obtained from the expression study of mRNA, the protein levels of IP3R1, VDAC1 and MCU were all upregulated after adipogenic differentiation but no change in the GRP75 protein (Figure 1B).

**FIGURE 2:**
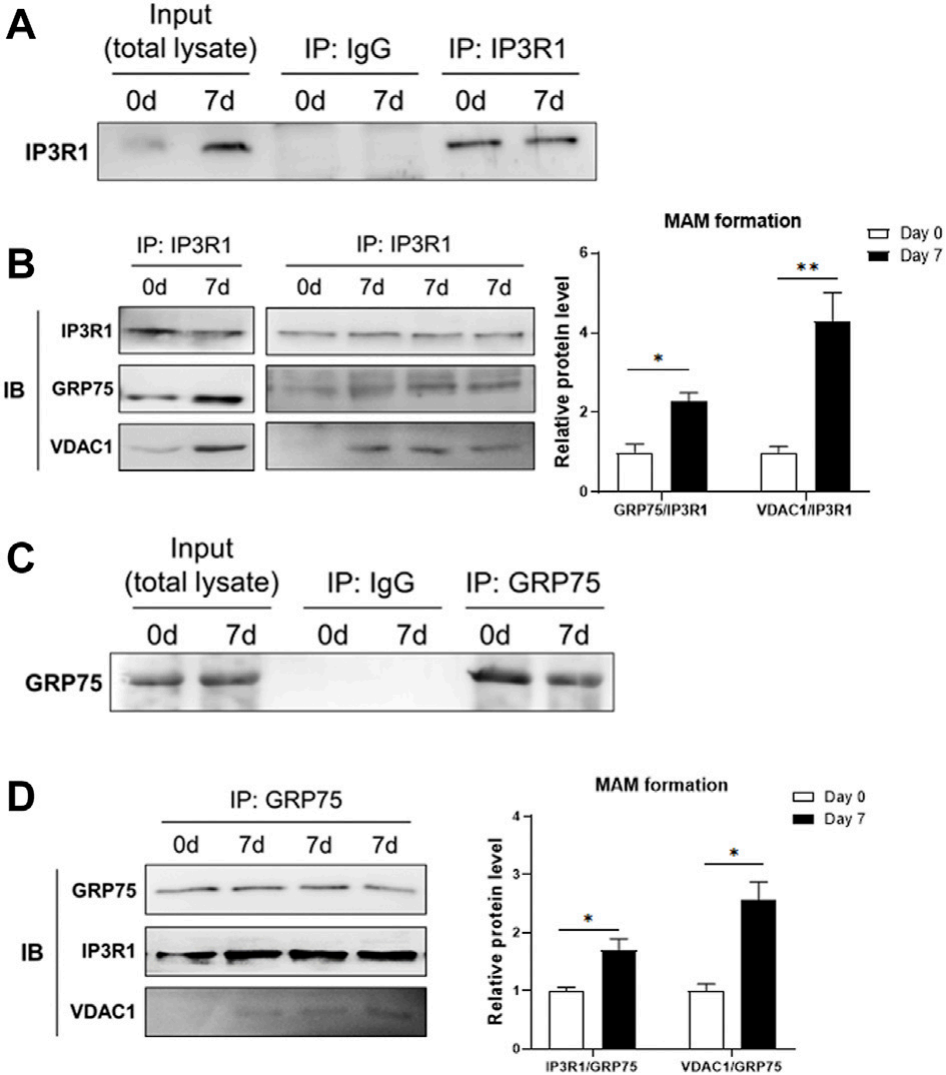
Increase of MAMs formation after adipogenic differentiation. **(A,B)** Protein lysates from 3T3-L1 preadipocytes (Day 0) and adipocytes (Day 7 after differentiation) were pulled down by an anti-IP3R1 antibody **(A)** and followed by detection of GRP75 and VDAC1 proteins using Western blot analysis (B, right panel). The MAMs formation was calculated by the ratio of interaction between IP3R1 and GRP75 and between IP3R1 and VDAC1, respectively (B, left panel). N = 3 in each group. **(C,D)** Protein lysates from 3T3-L1 preadipocytes (Day 0) and adipocytes (Day 7 after differentiation) were pulled down by an anti-GRP75 antibody **(C)** and followed by detection of IP3R1 and VDAC1 using Western blot analysis (D, right panel). The MAMs formation was calculated by the ratio of interaction between GRP75 and IP3R1 and between GRP75 and VDAC1, respectively (D, left panel). N = 3 in each group. Data are presented as means ± SEM.; *p < 0.05; **p < 0.01.

To check whether the formation of MAM structure is increased, we measured the interaction between IP3R1, GRP75 and VDAC1 using co-immunoprecipitation assay. We used anti-IP3R1 antibody to precipitate IP3R1 and its interacting proteins or protein complexes in the samples extracted before (day 0) and 7 days after adipogenic differentiation of 3T3-L1 preadipocytes. The results showed that after immunoprecipitation of IP3R1 with a limited amount of the primary antibody, an equal amount of IP3R1 was pulled down in the samples from day 0 and day 7 of differentiation although there was a high level of IP3R1 in the total lysate at day 7 (Figure 2A). We then used these IP3R1-immunoprecipitated samples to detect GRP75 and VDAC1 via immunoblotting (Figure 2B). We found that more GRP75 and VDAC1 proteins could be detected when the same amount of IP3R1 had been pulled down on day 7 compared to day 0 after differentiation (Figure 2B, left panel and Supplementary Figure S1). There are 2-fold or 4-fold increases in the interaction between GRP75 and IP3R1 or VDAC1 and IP3R1, respectively after 7 days of differentiation (Figure 2B, right panel). On the other hand, to further confirm the increase of MAMs formation during differentiation, we did the reciprocal co-immunoprecipitation. Similarly, after precipitating the same amount of GRP75 by its primary antibody (Figure 2C), 1.7-fold or 2.5-fold more IP3R1 and VDAC1 proteins could be pulled down on day 7 after differentiation, respectively (Figure 2D). Taken together, these findings suggest that MAMs formation (i.e., formation of IP3R1/GRP75/VDAC1 complex), is increased during adipogenic differentiation of 3T3-L1 preadipocytes.

### Disruption of mitochondria-associated endoplasmic reticulum membranes structure by GRP75 knockdown impairs adipogenic differentiation

**FIGURE 3:**
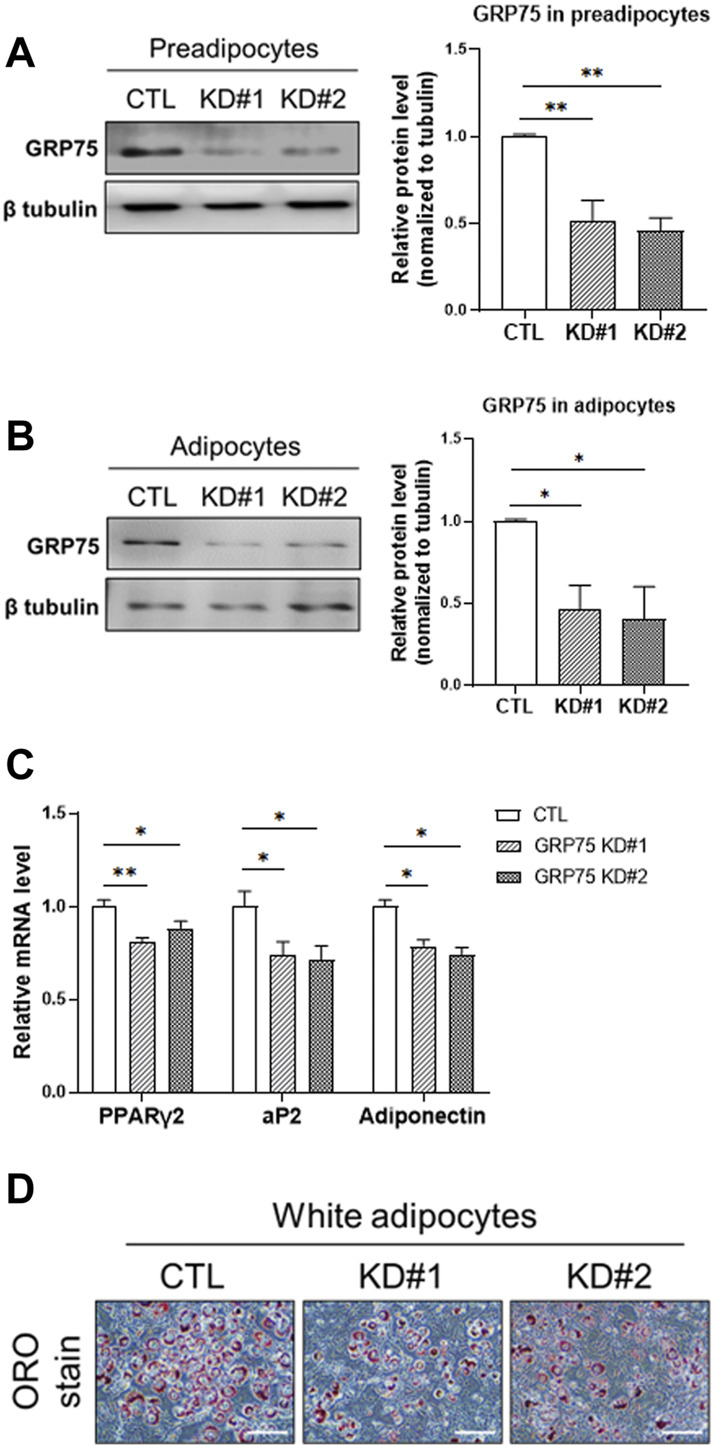
Impairment of adipogenic differentiation of 3T3-L1 preadipocytes with GRP75 knockdown. **(A)** The protein levels of GRP75 were determined in 3T3-L1 preadipocytes after delivering shRNA with scramble sequence (CTL) or two different sequences targeting to GRP75 (KD#1 and KD#2). The quantification of protein bands was shown in the left panel. N = 3 in each group. **(B,C)** The 3T3-L1 preadipocytes with CTL KD, GRP75 KD#1 and GRP75 KD#2 were induced to undergo adipogenic differentiation for 7 days. Protein levels of GRP75 **(B)** and mRNA levels of PPARγ2, aP2 and adiponectin **(C)** were determined by Western blot (N = 3 in each group) and RT-PCR (N = 6 in each group), respectively. **(D)** The 3T3-L1 preadipocytes with CTL KD, GRP75 KD#1 and GRP75 KD#2 were induced to undergo adipogenic differentiation for 7 days and were then stained by Oil red O. Scale bar = 500 μm. Data are presented as means ± SEM; *p < 0.05; **p < 0.01.

To determine the importance of MAMs formation in the differentiation and function of adipocytes, we disrupted the MAMs structure by knocking down the proteins involved in MAMs formation. Because IP3R1 and VDAC1 may have other exclusive roles in the function of ER and mitochondria, respectively, we decided to target GRP75, which is a linker between ER and mitochondria and its level was not changed during differentiation (Figure 1). We designed two different sequences of shRNA to knock down GRP75 in 3T3-L1 preadipocytes. After lentiviral delivery of shRNA, the protein levels of GRP75 were decreased 50% in 3T3-L1 preadipocytes by both GRP75 shRNA #1 and #2 compared to the scramble control shRNA group (Figure 3A). Most importantly, the knockdown of GRP75 was sustained for 7 days after adipogenic differentiation. The results showed that the protein level of GRP75 was declined 50% in mature adipocytes (Figure 3B). To determine the adipogenic differentiation capacity of 3T3-L1 with GRP75 KD, the adipocyte markers PPARγ2, aP2 (known as FABP4) and adiponectin were examined. The results revealed that the expression levels of these 3 genes were decreased after differentiation of 3T3-L1 preadipocytes with GRP75 KD (Figure 3C). In addition, we stained lipid droplets of adipocytes by Oil red O. In consistency with gene markers (Figure 3C), GRP75 deficiency led to the decreased formation of lipid droplets after 3T3-L1 differentiation (Figure 3D). These findings indicate that the disruption of MAMs structure by knock-down of GRP75 could impair the adipogenic differentiation of 3T3-L1 preadipocytes.

### Disruption of mitochondria-associated endoplasmic reticulum membranes structure leads to increase of ROS production

**FIGURE 4:**
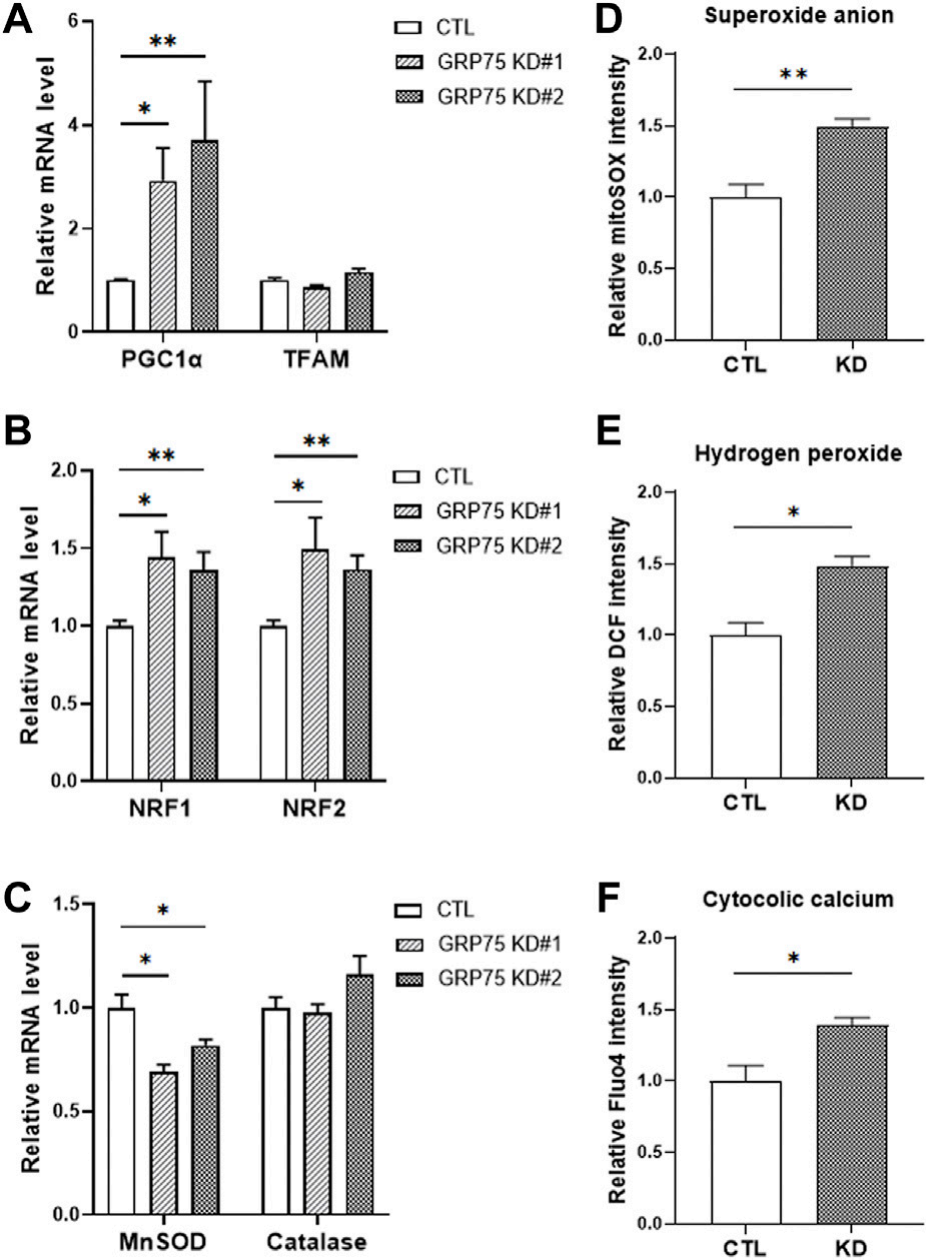
Accumulation of ROS in differentiated 3T3-L1 adipocytes with GRP75 knockdown. **(A–C)** The mRNA levels of mitochondrial biogenesis-related genes (PGC1α, TFAM, NRF1 and NRF2) and antioxidant enzymes (MnSOD and catalase) were determined by RT-PCR in 3T3-L1 preadipocytes with CTL KD, GRP75 KD#1 and GRP75 KD#2 7 days after differentiation. N = 6 in each group. **(D–F)** The intracellular levels of superoxide anions **(D)**, hydrogen peroxide **(E)** and cytosolic Ca^2+^ ions **(F)** were determined in 3T3-L1 preadipocytes with CTL KD, GRP75 KD#1 and GRP75 KD#2 7 days after differentiation using MitoSOX, DCF and Fluo-4 dyes, respectively. N = 4 in each group. Data are presented as means ± SEM; *p < 0.05; **p < 0.01.

Next, we examined whether disruption of MAMs structure affects the mitochondrial function during adipogenic differentiation. Surprisingly, adipocytes with GRP75 KD displayed upregulation of genes involved in mitochondrial biogenesis, such as PGC1α (Figure 4A). and NRF1/2 (Figure 4B). However, the increase of mitochondrial biogenesis did not lead to the increase of mitochondrial function in adipocytes. The adipocytes with GPR75 KD expressed less MnSOD (Figure 4C), which is a first-line antioxidant enzyme, leading to accumulation of intracellular reactive oxygen species (ROS) such as superoxide anions (Figure 4D) and hydrogen peroxide (Figure 4E). In addition, the cytosolic Ca^2+^ level was higher in mature adipocytes with GPR75 deficiency (Figure 4F). This may be due to the disruption of MAMs structure and mitochondrial dysfunction. Taken these findings together, disruption of MAMs formation triggered imbalance of redox status and Ca^2+^ dyshomeostasis and a compensatory increase of mitochondrial biogenesis may accelerate the defects due to the vicious cycle.

### Disruption of mitochondria-associated endoplasmic reticulum membranes structure leads to insulin insensitivity in 3T3-L1 adipocytes

**FIGURE 5:**
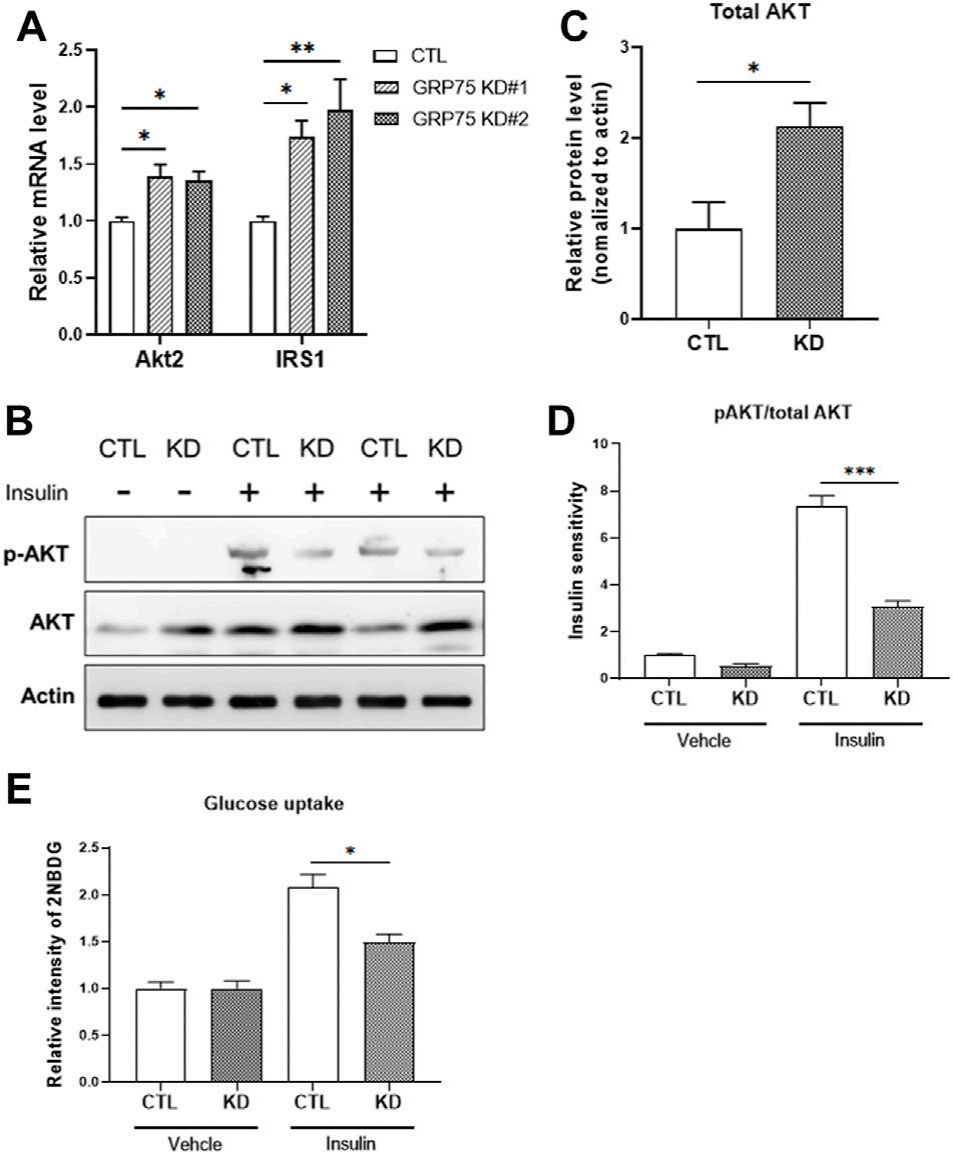
Insulin insensitivity of differentiated 3T3-L1 adipocytes with GRP75 knockdown. **(A)** The mRNA levels of genes involved in insulin signaling (Akt2 and Irs1) were determined in 3T3-L1 preadipocytes with CTL KD, GRP75 KD#1 and GRP75 KD#2 after differentiation. N = 6 in each group. **(B–D)** The 3T3-L1 preadipocytes with CTL KD and GRP75 KD#2 were induced to undergo adipogenic differentiation. The 7-days differentiated white adipocytes were treated with 100 nM of insulin and incubated for 30 min, and the cellular proteins were extracted to determine the levels of total AKT and phosphorylated AKT at serine 473 (p-AKT) **(B)**. The quantification of protein bands in total AKT of mature adipocytes **(C)**. Insulin sensitivity of adipocytes was determined by the ratio between p-AKT and total AKT **(D)**. N = 3 in each group. **(E)** Glucose uptake was determined by using 2-NBDG in differentiated CTL and GRP75 KD white adipocytes with or without insulin stimulation. N = 3 in each group. Data are presented as means ± SEM; *p < 0.05; **p < 0.01.

To answer whether the disruption of MAMs and mitochondrial dysfunction lead to functional defects of adipocytes, we determined insulin sensitivity in adipocytes with or without GRP75 KD by monitoring the activation of insulin signaling pathway. First, we observed that the mRNA expression levels of total AKT and IRS1 were upregulated at the basal state of adipocytes with GRP75 KD (Figure 5A). Similarly, the protein level of total AKT was also increased (Figures 5B,C). Since the phosphorylated AKT is the key to determine the activation of insulin signaling and glucose uptake (Batista et al., 2021), we then determined the phosphorylated AKT level and glucose uptake in CTL KD and GRP75 KD adipocytes upon insulin treatment. The results showed that, after insulin stimulation, AKT phosphorylation was dramatically induced in the control adipocytes (CTL KD), but such induction was impaired in the adipocytes with GRP75 KD (Figures 5B,D).

To determine whether less activation of AKT leads to impairment of glucose uptake, we used 2-NBDG, a fluorescent glucose analog, to measure glucose uptake in adipocytes. Although there is no difference in basal glucose uptake, GRP75 KD adipocytes displayed a decrease in insulin-stimulated glucose uptake (Figure 5E), which is consistent with impaired AKT activation (Figure 5D). These findings suggest that GRP75 deficiency impairs the activation of insulin signaling pathway, which in turn causes insulin insensitivity and affects glucose utilization in mature adipocytes.

### Disruption of mitochondria-associated endoplasmic reticulum membranes structure leads to defective thermogenic program and fuel utilization in brown adipocytes

For the regulation of energy metabolism, there are two types of adipose tissues in the human body, brown adipose tissue (BAT) and white adipose tissue (WAT). When compared with WAT, BAT contains more mitochondria and responds to the energy expenditure via thermogenesis. Thus, BAT displays a greater ability to modulate fatty acids and glucose homeostasis within the body (Cannon and Nedergaard, 2004; Shamsi et al., 2021). Since the mitochondrial function is more important for brown adipocytes than white adipocytes, we hypothesized that MAMs structure would play an essential role in thermogenesis of brown adipocytes.

**FIGURE 6:**
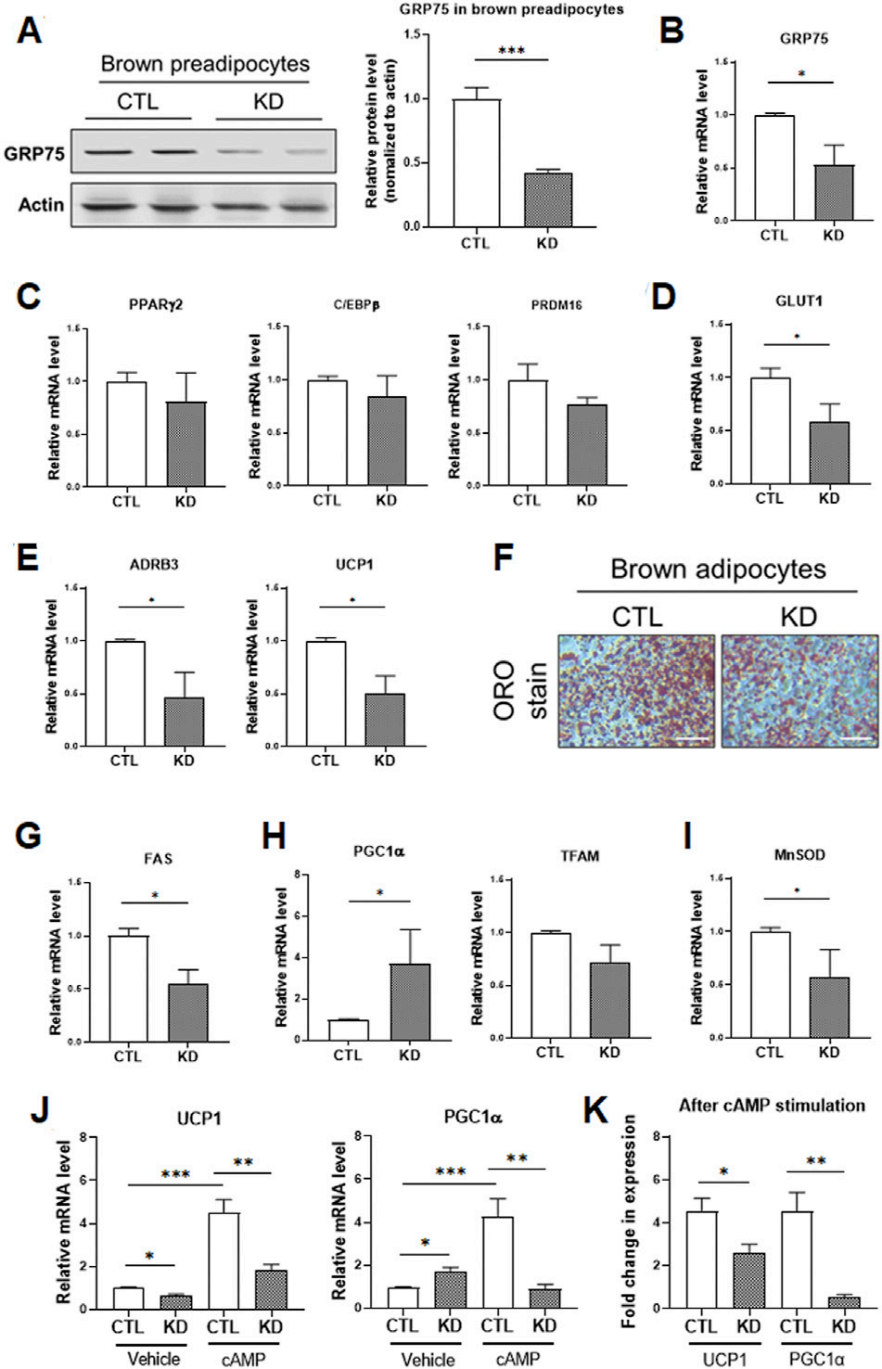
Impaired thermogenic program of differentiated brown adipocytes with GRP75 knockdown. **(A)** The protein levels of GRP75 were determined in differentiated brown preadipocytes after delivering shRNA with scramble sequence (CTL) or the sequence #2 targeting to GRP75 (KD). The quantification of protein bands was shown in the right panel. N = 4 in each group. **(B–E)** The brown preadipocytes with CTL KD or GRP75 KD were induced to undergo brown adipogenic differentiation. The mRNA levels of GRP75 **(B)** and genes involved in the regulation of adipogenic differentiation (PPARγ2, C/EBPβ and PRDM16; **(C)**, glucose uptake (Glut1; **(D)**, and thermogenesis (ADRB3 and UCP1; **(E)** were determined in 7-days differentiated brown adipocytes with CTL KD or GRP75 KD. N = 4 in each group. **(F)** Differentiated brown adipocytes with CTL and GRP75 KD were stained by Oil red O. Scale bar = 500 μm. **(G–I)** The mRNA expression levels of fatty acid synthase (FAS; **(G)** and genes involved in the regulation of mitochondrial biogenesis (PGC1α and TFAM; **(H)** and the antioxidant enzyme (MnSOD; **(I)** were measured in CTL and GRP75 KD brown adipocytes 7 days after differentiation of preadipocytes. N = 4 in each group. **(J,K)** The mRNA levels of UCP1 and PGC1α **(J)** in differentiated brown adipocytes were measured after treatment with vehicle or cAMP for 4 h. The fold changes of the mRNA expression levels after cAMP stimulation in CTL and GRP75 KD brown adipocytes are presented **(K)**. N = 4 in each group. Data are presented as means ± SEM; *p < 0.05; ***p < 0.001.

To address this issue, we knocked down GRP75 to disrupt MAMs structure in brown preadipocytes and investigated the genes involved in fuel utilization and thermogenic function. We established the brown preadipocytes with a 50% decrease of GRP75 protein by delivering sequence #2 of GRP75 shRNA (Figure 6A). After induction of differentiation of brown preadipocytes for 7 days, the decline of GRP75 expression (Figure 6B) was still maintained in the mature brown adipocytes. Loss of GRP75 did not cause any changes in the expression of genes regulating adipogenic differentiation such as PPARγ2, C/EBPβ and PRDM16 after brown adipocyte differentiation (Figure 6C). Intriguingly, GRP75 deficiency led to the downregulation of glucose utilization (GLUT1; Figure 6D) and thermogenic program (ADRB3 and UCP1; Figure 6E). In addition, decreased formation of lipid droplets revealed by Oil red O staining (Figure 6F) and decreased expression of fatty acid synthase (FAS; Figure 6G) indicate that fatty acid metabolism is impaired in brown adipocytes differentiated from GRP75 KD preadipocytes. Similar to the findings in 3T3-L1 white adipocytes, a loss of GRP75 in brown adipocytes resulted in the upregulation of the PGC1α expression (Figure 6H) to compensate for the declined mitochondrial function. However, a decrease the expression of antioxidant enzymes such as MnSOD (Figure 6I) may cause a vicious cycle to increase intracellular ROS levels like white adipocytes.

Mitochondrial biogenesis and UCP1 expression in brown adipocytes are further upregulated via β3-adrenergic receptor (β3-AR) signaling to promote thermogenic function during cold environment (Shamsi et al., 2021). In addition to the basal status, we investigated the response of brown adipocytes to β3-AR signaling. The differentiated brown adipocytes were treated with cAMP, a downstream second messenger of β3-AR signaling, for 4 h. UCP1 and PGC1α expression were significantly upregulated by 4.6 and 4.5-fold, respectively, in differentiated CTL brown adipocytes upon cAMP treatment (Figures 6J,K). In contrast, the inductions in UCP1 and PGC1α level in response to β3-AR signaling were decreased to 2.5 and 0.5-fold, respectively, in GRP75 KD brown adipocytes upon cAMP treatment (Figures 6J,K). All the findings indicated that disruption of MAMs structure led to mitochondrial dysfunction and not only impaired insulin response of white adipocytes but also thermogenic function of brown adipocytes.

## Discussion

This specical structure of MAMs is crucial for an accurate and efficient communication between the ER and mitochondria. The crosstalk includes the highly efficient transmission of physiological and pathological signals between the two organelles such as Ca^2+^ ions (Bononi et al., 2012). Due to the enrichment of Ca^2+^ handling proteins present in the MAMs, coupling at the ER-mitochondria interface is important for the maintenance of intracellular Ca^2+^ homeostasis and regulation of mitochondrial function and ROS formation required for cellular metabolism and cell survival. The MAMs have since been shown to be enriched in functionally diverse enzymes involved not only in lipid metabolism but also in glucose metabolism (Piccini et al., 1998; Stone and Vance, 2000). Defects in MAMs may have a role in the pathogenesis of various diseases such as Alzheimer’s disease and T2D (Walter and Hajnoczky, 2005; Leem and Koh, 2012; Hedskog et al., 2013). In a previous study, we showed that Cisd2, located on MAMs (Chen et al., 2009; Chang et al., 2012), interacts with Gimap5 and thereby modulates mitochondrial Ca^2+^ uptake for the maintenance of intracellular Ca^2+^ to regulate the differentiation and function of preadipocytes (Wang et al., 2014). This suggests that MAMs are involved in maintaining both function and integrity of ER and mitochondria, which may play an important role in the regulation of glucose homeostasis and insulin sensitivity.

In the present study, we further demonstrated that the disruption of MAMs structure by knocking down the linker protein, GRP75, could impair the differentiation and function of 3T3-L1 adipocytes, especially in the insulin signaling pathway. Similar to previous studies, decrease of mitochondrial function and overproduction of ROS in adipocytes with MAMs disruption may result in desensitized AKT activation and insulin insensitivity. In addition, we found that the compensatory upregulation of mitochondrial biogenesis together with an imbalance in the expression levels of antioxidant enzymes triggered the deleterious vicious cycle of ROS accumulation. However, scavenging of ROS by treatment of adipocytes with an antioxidant (N-acetyl cysteine) or by overexpression of antioxidant enzymes (e.g., MnSOD) warrants further investigation to confirm these speculations. Furthermore, we did not observe the upregulation of GRP75 expression during adipogenic differentiation although the MAMs formation was increased. Further study is warranted to elucidate whether other proteins facilitate the interactions between GRP75 and VDAC or between GRP75 and IP3R.

Although functions of white adipocytes such as insulin sensitivity and adipokine secretion play roles in the regulation of glucose/fatty acid homeostasis and energy metabolism (Wang et al., 2014; Ghaben and Scherer, 2019), emerging evidence has substantiated that brown adipocytes may play a more important role than white adipocytes (Bartelt and Heeren, 2014; Cypess, 2022). Compared to white adipocytes, brown adipocytes have greater capabilities in glucose/fatty acid uptake and utilization as well as stronger insulin response. Moreover, brown adipocytes also serve as an endocrine organ to regulate other tissues via secreted factors including proteins, lipids, metabolites, and exosomes (Shamsi et al., 2021). In addition to 3T3-L1 white adipocytes, we have also demonstrated the contribution of MAMs structure in the thermogenesis of brown adipocytes. In consistence with our results, it has been reported that the mitochondrial dysfunction and irregular formation of MAMs resulting from a defective proteasomal activity would damage the thermogenic function of BAT and led to obesity and glucose dyshomeostasis in mice (Bartelt et al., 2018). In addition, some proteins located in MAMs such as PERK (Kato et al., 2020) and Seipin (Combot et al., 2022) have been demonstrated to play a role in thermogenic function, Ca^2+^ homeostasis and glucose/lipid metabolism of brown adipocytes. In this study, we used GRP75, a structural protein required for the formation of MAMs, to strengthen the contribution of MAMs in the function of brown adipocytes.

There are many proteins resident in the MAMs structure, some are responsible for the regulation of mitochondrial function and others are required for ER function. In this study, we targeted the linker of the two organelles instead of the resident proteins in the ER or mitochondria. We have demonstrated the importance of MAMs formation in the maintenance of Ca^2+^ homeostasis and insulin sensitivity in white adipocytes by the dissociation between the ER and mitochondria via GRP75 deficiency. We also showed that the disruption of MAM structure impaired thermogenic function and fatty acid metabolism of brown adipocytes. However, further studies are required to unravel the mechanisms between MAM formation and brown adipocyte function. In conclusion, our findings of this study suggest that the MAMs structure is crucial for the functions of ER and mitochondria and its disruption in adipocytes would lead to insulin insensitivity and T2D due to the overproduction and inefficient disposal of intracellular ROS.

## Data Availability

The original contributions presented in the study are included in the article/Supplementary Material, further inquiries can be directed to the corresponding author.
