# Supplementary figures and images for: Disruption of mitochondria-associated ER membranes impairs insulin sensitivity and thermogenic function of adipocytes
Source: Front Cell Dev Biol. 2022 Sep 9;10:965523. doi: 10.3389/fcell.2022.965523 (PMC9504280; doi:10.3389/fcell.2022.965523)

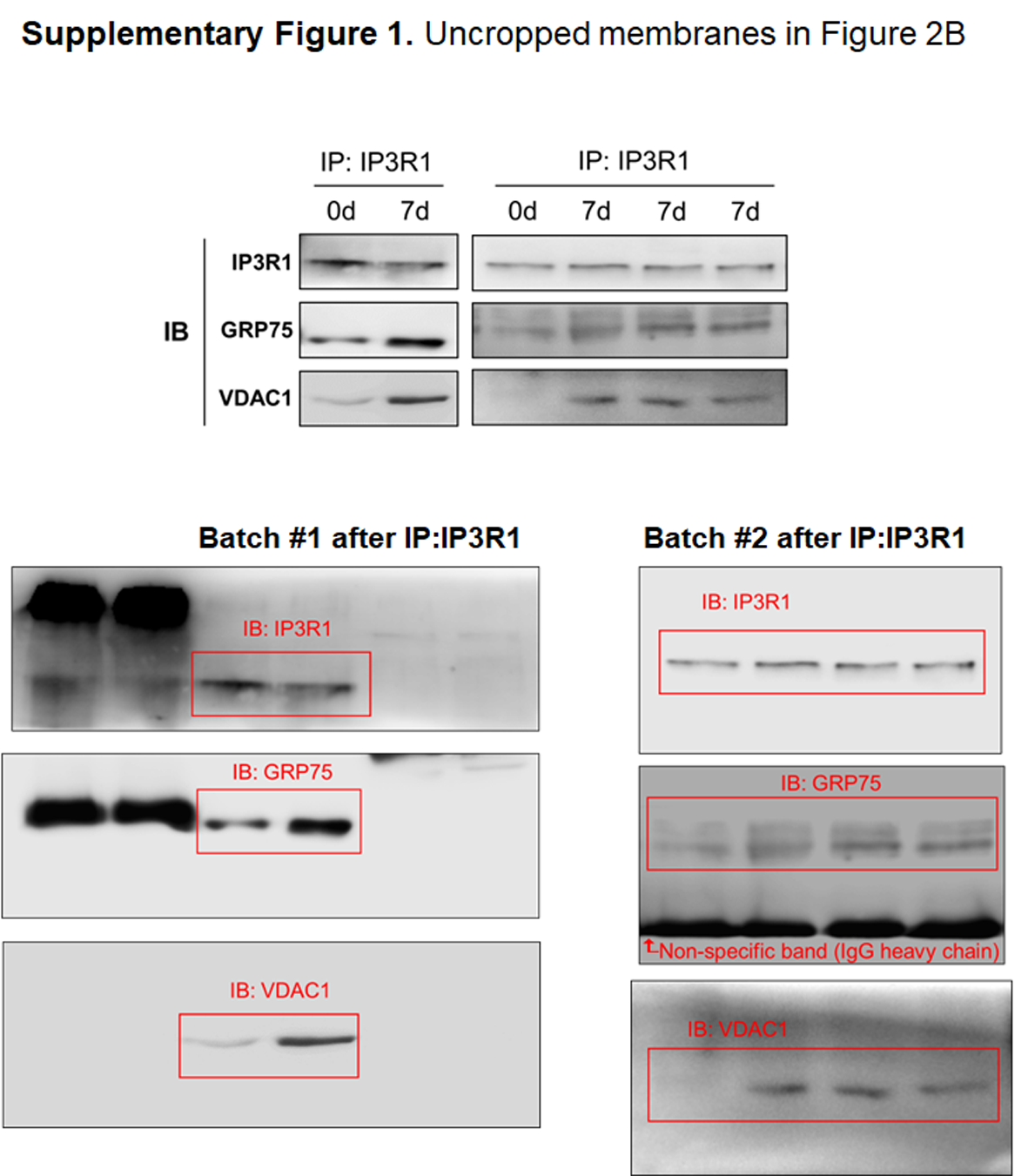

Supplement: Supplementary file 1 [file Image1.TIF]
